# The Effect of Gender-Affirming Medical Care on the Vaginal and Neovaginal Microbiomes of Transgender and Gender-Diverse People

**DOI:** 10.3389/fcimb.2021.769950

**Published:** 2022-01-21

**Authors:** Yonah Krakowsky, Emery Potter, Jason Hallarn, Bern Monari, Hannah Wilcox, Greta Bauer, Jacques Ravel, Jessica L. Prodger

**Affiliations:** ^1^ Division of Urology, Department of Surgery, Women’s College Hospital and Mount Sinai Hospital, University of Toronto, Toronto, ON, Canada; ^2^ Transition Related Surgery, Department of Surgery, Women’s College Hospital, University of Toronto, Toronto, ON, Canada; ^3^ Department of Epidemiology and Biostatistics, Schulich School of Medicine & Dentistry, Western University, London, ON, Canada; ^4^ Program in Molecular Medicine, University of Maryland School of Medicine, Baltimore, MD, United States; ^5^ Department of Microbiology and Immunology, Schulich School of Medicine & Dentistry, Western University, London, ON, Canada; ^6^ Institute for Genome Sciences, University of Maryland School of Medicine, Baltimore, MD, United States; ^7^ Department of Microbiology & Immunology, University of Maryland School of Medicine, Baltimore, MD, United States

**Keywords:** vagina, neovagina, transgender, gender diverse, microbiome, bacterial vaginosis

## Abstract

Transgender and gender diverse individuals may seek gender-affirming medical care, such as hormone therapy or surgery, to produce primary and/or secondary sex characteristics that are more congruent with their gender. Gender-affirming medical care for transmasculine individuals can include testosterone therapy, which suppresses circulating estrogen and can lead to changes in the vaginal epithelium that are reminiscent of the post-menopausal period in cisgender females. Among transfeminine individuals, gender-affirming medical care can include vaginoplasty, which is the surgical creation of a vulva and neovaginal canal, commonly using penile and scrotal skin. The effect of gender-affirming medical care on the vagina of transmasculine individuals and on the neovagina of transfeminine individuals is poorly characterized. This review summarizes what is known of the epithelium and local microbiota of the testosterone-exposed vagina and the neovagina. We focus on potential pathogens and determinants of gynecological health and identify key knowledge gaps for future research.

## Introduction

Transgender and gender diverse (TGD) individuals have a gender identity that is incongruent with the sex/gender they were assigned at birth. Other key terminology used in this review is listed in **Table 1**. It is estimated that 0.2-0.5% of the North American adult population is TGD (Conron et al., 2012; Scheim and Bauer, 2015; Helman et al., 2016; Meerwijk and Sevelius, 2017; Zucker, 2017; Jaffray, 2020). Many TGD individuals seek gender-affirming medical care, such as hormone therapy or surgery, to produce primary and/or secondary sex characteristics that are more congruent with their gender. Gender-affirming medical care can be a critical and life-saving step for many: a meta-analysis of 28 studies reported significant improvements in gender dysphoria (80% of individuals), psychological symptoms (78%), quality of life (80%), and sexual function (72%) for those who underwent gender-affirming medical care with hormones and/or surgery (Murad et al., 2010).

**Table 1:** Key terminology.

Gender	Social characteristics differentiating women/girls, men/boys, and gender-diverse people, including gender identity, gender expression, gender roles, and institutional gender.
Sex	Biological characteristic differentiating females, males, and intersex people, including chromosomal, anatomical, and physiological factors.
Transgender (trans)	A person whose gender identity does not align with that associated with their sex assigned at birth. *Adjective*
Cisgender (cis)	A person whose gender identity aligns with that associated with their sex assigned at birth. *Adjective*
Transmasculine (tM)	An individual assigned female at birth who identifies as male, man/boy, masculine, non-binary and/or something other than a woman/girl. *Adjective*
Transfeminine (tF)	An individual assigned male at birth who identifies as female, woman/girl, feminine, non-binary and/or something other than a man/boy. *Adjective*
Transgender and gender diverse	An inclusive term for those who have a gender identity that is incongruent with the sex/gender they were assigned at birth. This can include many different identities such as transmasculine, transfeminine, trans, non-binary, two-spirit, genderqueer, agender, and many others.
Gender dysphoria (generic)	Discomfort or distress caused by a discrepancy between an individual’s experienced/expressed gender and their assigned gender and/or primary or secondary sex characteristics.
Gender dysphoria (diagnostic label)	A diagnostic label used when an individual meets the full set of DSM-5* criteria for gender dysphoria.
Gender-affirming medical care	The process through which individuals alter their primary and/or secondary sex characteristics to align with their sense of gender identity through medical treatment.
Vaginoplasty	The surgical construction of a vaginal cavity. Vaginoplasty is a gender-affirming surgery that is undergone by some transfeminine individuals.
Full-depth vaginoplasty	A vaginoplasty surgery that creates a neovagina with sufficient depth, approximately 9cm or more
Vulvaplasty/Zero-Depth Vaginoplasty	A vaginoplasty surgery that does not create a vaginal cavity.
Neovagina	A term used to refer to the vagina that has been surgically constructed through vaginoplasty.
Vaginectomy	The surgical closure of the vaginal cavity.

*DSM-5, Diagnostic and Statistical Manual of Mental Disorders. DSM–5 is the standard classification of mental disorders used by mental health professionals in the United States.

Although not all TGD individuals identify as a binary gender (either a man or woman), gender-affirming medical care, including exogenous sex hormones, hormone blockers and/or surgeries, is sometimes used by TGD people to either masculinize or feminize the body. In this respect, we use the terms transfeminine (tF) for individuals assigned male at birth but who do not identify as male and may undergo feminizing gender-affirming medical care, and transmasculine (tM) for individuals who were assigned female at birth but do not identify as female and may undergo masculinizing gender-affirming care. Likewise, we use the terms cis female (cF) for individuals who were assigned female at birth and identify as female and the term cis male (cM) for individuals who were assigned male at birth and identify as male.

Hormone therapy is a common component of gender-affirming medical care for TGD individuals. The 2015 US Transgender Survey (USTS) reported that 49% of TGD individuals have received hormone therapy and a further 29% desired it (James et al., 2019). Feminizing hormone therapy usually consists of testosterone suppression, estrogen (estradiol) and occasionally progestin; these promote the development of secondary sex characteristics such as breasts, body fat redistribution, and softening of the skin, among others (T'Sjoen et al., 2019). Masculinizing hormone therapy usually consists of testosterone, which promotes secondary sex characteristics such as suppression of menstrual cycles, voice deepening, facial and body hair growth, body fat redistribution, and clitoral enlargement (T'Sjoen et al., 2019).

Genital surgery may also be a component of gender-affirming medical care. The 2015 USTS reports that 10% of tF individuals had completed vaginoplasty (the surgical creation of a neovaginal cavity) and a further 45% of respondents reported wanting to have the procedure in the future (James et al., 2019). Vaginectomy and other masculinizing gender-affirming genital surgeries are rarer, but hysterectomy is relatively common. The USTS reported 8% of tM respondents had undergone hysterectomy with a further 44% desiring to have this surgery, but only 2% had undergone metoidioplasty and/or phalloplasty (James et al., 2019). In Ontario, Canada, 2009-2010 data estimate even higher proportions, with 15% of tF Ontarians having completed vaginoplasty and 13% of tM Ontarians having undergone hysterectomy (Scheim and Bauer, 2015).

We will employ the commonly used term “neovagina” to refer to vaginas that are surgically created by vaginoplasty, and “vagina” to refer to vaginas that were present at birth. Furthermore, we will refer to the present-at-birth vaginas of those taking testosterone therapy (tM individuals) as *testosterone dominant vaginas* (TDV) and vaginas of reproductive-aged individuals who are not taking testosterone therapy (including both cF and TGD individuals not on testosterone therapy) as *estrogen dominant vaginas* (EDV). The field of transgender medicine is relatively new, and little is known of the effects of testosterone therapy on the TDV nor of estrogen therapy on the neovagina, but it is clear that both genital microenvironments are distinct from the comparatively better studied EDV. As social acceptance increases and access to gender-affirming medical care continues to improve, the number of TGD individuals who will need tailored gynecological care is increasing. The provision of inclusive healthcare is necessary to achieve optimal health and reduce inequities experienced by TGD communities (American College of Obstetricians and Gynecologists’ Committee on Gynecologic Practice and American College of Obstetricians and Gynecologists’ Committee on Health Care for Underserved Women, 2021).

This review summarizes what is known of the epithelium and local microbiota of the TDV and the neovagina. We focus on potential pathogens and determinants of gynecological health and identify key knowledge gaps. Our review raises more questions than it provides answers, underscoring an urgent need for research on these distinct genital microenvironments.

## Microenvironment of the Testosterone Dominant Vagina

### Estrogen Shapes the Vaginal Microenvironment

The vaginal mucosa is a stratified squamous epithelium that undergoes continuous renewal through proliferation of basal cells, and thus newly formed epithelial cells are pushed outward towards the lumen by the subsequent cell generations. As basal cells lose contact with the basement membrane, they begin to differentiate, expressing cytokeratins K4/K13 and K1/K10 (which form intermediate filaments, and whose expression is organ-specific), to eventually reach full maturation in the superficial layers (Waseem et al., 1998). Maturation of vaginal epithelial cells is regulated by estrogen, which promotes epithelial cell proliferation and thus increases thickness of the epithelium (Ayehunie et al., 2015). Estrogen also promotes the production of glycogen, a glucose polysaccharide, by vaginal epithelial cells (Cruickshank, 1934; Anderson et al., 2014). Cell-cell junctions are lost during cellular maturation and the loosely connected, glycogen-rich cells of the superficial layer are readily shed into the vaginal lumen. Glycogen from shed epithelial cells is catabolized by both human and bacterial α-amylases in the vaginal lumen to smaller polymers that are a preferred carbon source of beneficial *Lactobacillus* spp., but also of non-desirable anaerobic bacteria (Mirmonsef et al., 2014; Spear et al., 2014; van der Veer et al., 2019; Nunn et al., 2020). Lactobacilli then metabolize glycogen-derived polymers into lactic acid, which reduces the pH of the vaginal lumen, favoring the proliferation of lactobacilli and inhibiting the growth of pathogenic organisms such as *Neisseria gonorrhoeae*, *Chlamydia trachomatis* and those associated with bacterial vaginosis (BV) (Graver and Wade, 2011; O'Hanlon et al., 2011; O'Hanlon et al., 2013; Gong et al., 2014; Mirmonsef et al., 2014; Breshears et al., 2015; Mirmonsef et al., 2016; Nardini et al., 2016; Edwards et al., 2019). In addition to reducing the pH, *Lactobacillus* spp. also prevent colonization by pathogens through the production of bacteriocins and biosurfactants (Valore et al., 2002). In the absence of *Lactobacillus* spp., the EDV is colonized by a diverse set of strict and facultative gram-positive and gram-negative anaerobes (e.g., *Atopobium*, *Prevotella*, *Gardnerella*) (Ravel et al., 2013; France et al., 2020; Holm et al., 2020; Turpin et al., 2021). Communities dominated by diverse anaerobes are reminiscent of BV, which is characterized by abnormal discharge, itching, malodour, and an elevated pH (Eschenbach et al., 1988; Schwebke et al., 1996; Redelinghuys et al., 2020). Even in the absence of symptoms, vaginal microbiomes deficient in lactobacilli (“molecular BV”) are associated with impaired epithelial maturation, increased mucosal inflammation, changes in epithelial barrier function, increased susceptibility to sexually transmitted infections (chlamydia, gonorrhea, HIV and HPV, among others), and reproductive risks (Leitich et al., 2003; Wiesenfeld et al., 2003; Brotman et al., 2010; Arnold et al., 2016; Zevin et al., 2016; Joag et al., 2019; Tamarelle et al., 2019; O'Hanlon et al., 2020).

### Effect of Testosterone Therapy on the Vaginal Microenvironment

Testosterone therapy is highly effective in allowing TGD individuals to develop the secondary sex characteristics associated with masculinity, but the suppression of estrogen (**Table 2**) can induce epithelial thinning reminiscent of the estrogen-deprived post-menopausal cF vagina (Baldassarre et al., 2013).

**Table 2:** Expected Serum Hormone Ranges of Populations of Interest (Leinung et al., 2018; Greene et al., 2019; Greene et al., 2020; M. C. Laboratories, 2021).

Population	Estrogen (pg/ml)	Progesterone (ng/ml)	Testosterone (ng/dl)
Cis Women, Reproductive-Age	15 – 350	0.9 – 24	8 – 60
Cis Women, Post-Menopausal	<10	≤0.2	8 – 60
Cis Men	10 – 40	≤0.2	240 – 950
Transmasculine	29 – 51	0.3 – 0.7	320 – 630
Transfeminine	207	Not available	9 – 34

In the absence of estrogen, vaginal epithelial cell proliferation slows, and the epithelium becomes thinner and more fragile, leading to dryness, irritation, and dyspareunia (pain during intercourse) (Pessina et al., 2006; Perrone et al., 2009; Baldassarre et al., 2013). Decreased estrogen in the post-menopausal period is also associated with reduced glycogen deposition and, combined with reduced epithelial proliferation and turnover, results in a marked reduction in the availability of free glycogen in the mucosa (Mirmonsef et al., 2015). Potentially due to reduced glycogen availability, the vaginal microbiome post-menopause is significantly less likely to be dominated by lactobacilli, and instead is more likely to be colonized with a unique, diverse microbiota. This microbiota has some overlap with molecular BV in the EDV (e.g., *Prevotella*, *Gardnerella*, *Dialister*), but is clearly distinct, with higher abundance and prevalence of genera such as *Streptococcus*, *Corynebacterium*, *Finegoldia*, *Peptoniphilus*, *Anaerococcus*, and *Bifidobacterium* and lower abundance of BV-associated genera *Atopobium*, *Sneathia*, and *Megasphaera* (Brotman et al., 2014; Mirmonsef et al., 2014; Shen et al., 2016; France et al., 2020). The importance of estrogen in shaping the vaginal microbiome is underscored by the effects of local or systemic estradiol-based hormone replacement therapy post-menopause, which restores lactobacilli dominance, decreases vaginal pH, and alleviates symptoms of vaginal fragility (Brotman et al., 2014; Shen et al., 2016). In TDV, testosterone therapy has been shown to thin the epithelium, with histological evaluation revealing lowered cell proliferation, loss of the intermediate and superficial strata, and reduced glycogen deposition compared to pre-menopausal EDV (Baldassarre et al., 2013). Recently published data indicates that TDV tissue has elevated levels of inflammation, edema, collagen fibrosis, and granulation tissue (Schardein et al., 2021). Transmasculine individuals on testosterone therapy frequently experience symptoms of vaginal atrophy similar to those of the post-menopausal state, including dryness, irritation, bleeding with vaginal penetration (sex or medical examination), and dyspareunia (Peitzmeier et al., 2014; Potter et al., 2015). These symptoms can have a substantial impact on quality of life, and as such some tM individuals opt for topical estriol or estradiol administered directly to the vaginal mucosa *via* cream, a ring, or tablets (Santen, 2015). While local estrogen-based therapy to treat vaginal atrophy is included in multiple trans care guidelines, the efficacy of this approach has not been documented in tM (Deutsch, 2016; Obedin-Maliver and de Haan, 2017; Bourns, 2020).

### Microbiome of the Testosterone Dominant Vagina

To our knowledge, there has been a single study describing the TDV microbiome (Winston McPherson et al., 2019). In this study, 16S rRNA gene sequencing was used to identify the proportional abundances of bacterial species in TDV swabs collected from 28 tM individuals on testosterone therapy. Only 3/28 TDV had a *Lactobacillus*-dominated microbiota; instead, the majority of TDVs had microbiota composed of a diverse set of anaerobic taxa, more like microbiota observed in post-menopause cF than molecular BV (i.e., containing *Anaerococcus*, *Corynebacterium*, *Finegoldia*, *Peptoniphilus*, *Streptococcus*, in addition to *Prevotella*, *Dialister*, *Gardnerella*, but with low abundance of *Atopobium*, *Sneathia*, *Megasphaera*). Despite sharing some similarities with the post-menopausal vagina, the microbiota observed in the TDV was also clearly distinct, with higher abundance of *Campylobacter*, *Fusobacterium*, *Parvimonas*, and *Porphyromonas*, indicating testosterone augmentation may influence the composition of the vaginal microbiota beyond that of estrogen reduction. It is notable that, of the three individuals who had a *Lactobacillus-*dominated microbiota, two were prescribed topical estradiol (a total of 4 individuals in the study had been prescribed topical estradiol to treat symptoms of vaginal atrophy). Despite limited statistical power in this relatively small study, the correlation between vaginal estrogen therapy and presence of a *Lactobacillus*-dominated microbiota was statistically significant (p=0.045). This study provides an important first assessment of the TDV microbiota, suggesting it is distinct from the microbiota observed post-menopause, and that *Lactobacillus-*dominated microbiota are rare.

### Knowledge Gaps

Despite the progress made in recent years, several key gaps remain in the literature. Additional studies are warranted to confirm and expand on the seminal publication by McPherson et al. (Winston McPherson et al., 2019). Larger longitudinal studies, including following TGD participants through the initiation of testosterone therapy and studies of individuals who have been on testosterone for decades, would provide detailed information on the specific effects of testosterone therapy. Additionally, many TGD individuals interrupt testosterone therapy to become pregnant (Obedin-Maliver and Makadon, 2016). Molecular BV in cF individuals is associated with higher risk of serious reproductive risks (Leitich et al., 2003); it is unknown how the unique microbiota of the TDV may influence reproductive outcomes. Finally, to inform appropriate clinical treatment guidelines for the medical care of TGD individuals, new studies should focus on relating microbiota composition and function to symptomology, immune status, and local energy sources available to microbes.

Another important knowledge gap is whether locally administered vaginal estrogen therapy could be used to treat vaginal atrophy and promote *Lactobacillus* colonization in tM individuals without interfering with the masculinizing effects of testosterone. Local estrogen therapy is commonly recommended to treat vaginal atrophy post-menopause (Kaur et al., 2020; Shim et al., 2021), and, in low doses, this therapy can alleviate symptoms without substantially increasing systemic estrogen levels (Lethaby et al., 2016). The effect of local estrogen therapy on systemic levels will likely be dependent on characteristics of the vaginal microenvironment [reviewed in (Santen, 2015)], including vaginal epithelial thickness and the local microbiome. Given the potential benefits, research is warranted to assess the acceptability and efficacy of locally administered vaginal estrogen therapy in TGD individuals on testosterone.

Third, our ability to study the effect of testosterone therapy on vaginal and cervical epithelia have been hampered by lack of appropriate model systems. Monolayers of cells in submerged culture do not replicate the stratified epithelium of the vagina and do not provide an appropriate environment for the culture of vaginal bacteria, while animal models do not replicate the relationship between the human vagina and its unique *Lactobacillus*-dominated microbiota (Couri et al., 2012; Barfod et al., 2013; Cassone and Sobel, 2016). Three-dimensional air-liquid interface cell culture allows for stratification of cultured vaginal epithelial cells and provides a more relevant environment for vaginal bacteria (Lee et al., 2016; Zhu et al., 2017). The utility of this model in delineating the impact of testosterone therapy on the cervicovaginal epithelium warrants further investigation.

## The Neovaginal Microenvironment

### Vaginoplasty and the Neovaginal Epithelium

Penile inversion vaginoplasty is the gold standard surgical technique of feminizing genital surgery (Bizic et al., 2014; Horbach et al., 2015; Buncamper et al., 2016; Dreher et al., 2018; Bustos et al., 2021; Moises da Silva et al., 2021). This surgery was first introduced in the early 1900’s and has undergone various permutations in search of the optimal outcome (Horbach et al., 2015). The ideal outcome of this surgery is a concordant vulvar anatomy, moist and hairless vagina with sufficient depth and width for types of penetration desired (if any), erogenous sensation, and requiring minimal maintenance (Garcia et al., 2020). This surgery requires many surgical steps; orchiectomy, clitoroplasty, penile de-gloving and resection of the corpora cavernosa, shortening and splaying of the urethra, surgical dissection of the space between the bladder and the rectum and the inversion of the flap of preserved penile tube skin and placement into this space. Frequently, the penile tube skin alone is insufficient to generate a vaginal canal with adequate depth and additional skin grafts, most commonly from scrotal skin, are used to augment length (Selvaggi et al., 2005; Goddard et al., 2007; Goddard et al., 2007; Dy et al., 2018). Hair from scrotal grafts is typically removed intraoperatively by thinning the scrotal graft and cauterizing visible follicles. The proportion of scrotal/penile skin used to line the canal is influenced by the amount of tissue present and the depth of the pelvic dissection and is not well identified in the literature.

Alternative procedures are used to create sufficient depth in the case that the penile and scrotal skin is insufficient. These techniques are generally recommended for revision surgeries because of their accompanying risks and complications. Bowel pedicle flaps (i.e., sigmoid colon, ileum, and transverse colon), regional and isolated skin flaps (i.e., thigh or lower abdomen), peritoneal flaps, and the incorporation of urethral mucosa into the inverted skin flap are all identified in the literature. Despite the multiple options available to line the neovagina, the penile scrotal flap is by far the most commonly used (Horbach et al., 2015; Buncamper et al., 2016), with the rectosigmoid colon bowel flap as the most common alternative procedure (Horbach et al., 2015).

Understanding the physiology and structure of neovaginal canals made with different tissues is essential, because features of the neovaginal epithelium are very likely to shape microbiome composition and function, and thus are expected to have a substantial impact on gynecological health and quality of life. It is well established that environment (i.e., local levels of oxygen, humidity, and environmental exposures) shapes the local microbiome, unambiguously demonstrated by the effect of penile circumcision on the composition of the coronal sulcus microbiota (Liu et al., 2013). Elimination of the foreskin increases water loss and oxygen tension on the coronal sulcus, which decreases the abundance of many strict anaerobes (including *Prevotella, Finegoldia, Peptotreptococcus, Peptoniphilus, Porphyromonas, Dialister, Murdochiella*, and *Negativococcus*) and increases aerobes and facultative anaerobes (e.g., *Corynebacterium* and *Staphylococcus*). However, in addition to the environment, characteristics of the epithelium itself can have a dramatic influence on the local microbiome. For example, during puberty estrogen induces changes in the vaginal epithelium, increasing thickness and glycogen content, which is associated with a dramatic shift in the local microbiome, from one dominated by a diverse set of strict and facultative anaerobes to *Lactobacillus* domination and an acidic pH (<4) (Schaller, 1990).

Similar to the vaginal epithelium, penile skin is a stratified squamous epithelium that is constantly renewing through proliferation of basal cells. However, the epithelial layer of penile skin is thinner than that of the pre-menopausal EDV (100 vs 300μm) (Baldassarre et al., 2013; Carias and Hope, 2019), expresses different cytokeratins (K5/K14 in intermediate layers followed by K1/10 in superficial layers), and has a soft-cornified outer layer (15-20μm thick) comprised of terminally differentiated keratinocytes that have undergone programmed cell death, lack nuclei and organelles, and are filled with keratin bundles (Stankler and Walker, 1976; Dinh et al., 2012). Fully mature skin keratinocytes limit water loss by extruding lamellar bodies to form an intercellular lipid envelope, and provide mechanical integrity through specialized cell junctions called corneodesmosomes. Desquamation of skin corneocytes is controlled by degradation of corneodesmosomes and this process frees keratin and fatty acids that are nutrient sources for bacteria and shape the microbiota (Gupta and Ramnani, 2006; Houben et al., 2008; Bragulla and Homberger, 2009; Grice and Segre, 2011). This contrasts with the outermost layer of the EDV, which has loosely connected superficial cells filled with glycogen, which when shed release glycogen and promote colonization with lactobacilli (Pask et al., 2008; Bragulla and Homberger, 2009; Menon et al., 2012; Anderson et al., 2014; Tjernlund et al., 2015). The sigmoid colon epithelium is also highly distinct from that of the EDV and the penis. It is a single-layer columnar epithelium expressing the cytokeratin pair K8/K18 and containing highly specialized epithelial cells such as goblet cells (producing mucus) and Paneth cells (producing antimicrobial peptides), among others.

Very little is known of the influence of surgical invagination and exogenous estrogen on the differentiation pattern of epithelial cells in the neovagina. It is possible that reduced water loss from surgical invagination may alter the epithelial differentiation patterns of once-penile skin, for example, through reduced lamellar body and corneodesmosome formation, resulting in an epithelial surface more similar to the EDV. However, while vaginal epithelial cells have the ability to differentiate into corneocytes in response to hormonal or mechanical signals, potentially owing to their expression of both K4/K13 (typical of non-cornified stratified epithelia) and K1/K10 (Schaller, 1990; Schaller and Genz, 1990; Schaller et al., 1993; Bragulla and Homberger, 2009), epithelial cells derived from skin and the sigmoid colon contain low levels of glycogen and do not express K4/K13 (Menon et al., 2012). One small study has examined the microstructure of the neovaginal epithelium created from penile skin (n=9) (Dekker et al., 2007). This study observed that cornification was reduced but not lost, and no glycogen production was observed, even among the three participants who had vaginoplasty more than nine years prior and had been receiving estrogen hormone therapy for >11 years. The absence of glycogen and retention of cornification suggest that it would be difficult for the neovagina to support a *Lactobacillus-*dominated microbiota. While a neovagina constructed from entirely penile skin and one that includes sigmoid colon may have similar environmental exposures (oxygen levels, estrogen levels, etc.), factors such as residual cornification or the presence of goblet cells producing mucus may dictate what bacteria colonize the neovagina microenvironment, and what bacteria are beneficial vs. pathogenic. Therefore, different treatment courses may be required for individuals suffering from neovaginal symptoms, depending on the tissue used to create their neovaginal canal. Altogether, our knowledge of the epithelia used for vaginoplasty does not support the notion that an optimal neovaginal microenvironment would comprise *Lactobacillus* spp. and an acidic pH.

### The Neovaginal Microbiome

Despite the enormous impact of the vaginal microbiome on cF sexual and reproductive health, there have been few reports of the microbiota colonizing the neovagina and there is no knowledge of what microbiota are optimal vs. associated with inflammation, symptoms, and STI risk. Until recently, data on the microbiota colonizing the neovagina were limited to case reports and small studies that used limited culture-based detection methods or targeted PCR to detect the presence of specific species of interest (classic STI pathogens or *Lactobacillus* spp.) (Bodsworth et al., 1994; Haustein, 1995; Weyers et al., 2009; Weyers et al., 2010; de Haseth et al., 2018; Radix et al., 2019). These assays fail to capture the vast majority of species present and provide no information on the composition or structure of bacterial communities in the neovagina. Recently, one study by Birse et al. (2020) used a combination of proteomics and 16S rRNA gene sequencing to examine the neovaginal microbiome in five tF individuals, four of whom had penile inversion vaginoplasty and one whom had sigmoid vaginoplasty (median 10 years post-vaginoplasty, range 4-36 years). While the small sample size limits the ability to draw conclusions, this important study is the first examination of the neovaginal microbiome and hints at interesting hypotheses (**Table 3**). Of the four tF individuals who underwent penile inversion vaginoplasty, *Lactobacillus* was detected in one individual at low abundance. Instead, highly prevalent genera in the penile-skin lined neovagina included *Prevotella* and *Peptostreptococcus* (both also prevalent in molecular BV of the EDV, the post-menopausal vagina, and the TDV); *Peptoniphilus* and *Corynebacterium* (also prevalent in the post-menopausal vagina and the TDV); and *Porphyromonas* and *Campylobacter* (also prevalent in the TDV). Interestingly, these genera are also highly abundant/prevalent within the foreskin fold of the uncircumcised penis. The skin under the foreskin fold is usually colonized with a diverse set of strict and facultative anaerobes, the most prevalent and abundant being *Prevotella*, *Porphyromonas*, and *Peptoniphilus*, while the circumcised penis is usually dominated by *Corynebacterium* (Price et al., 2010; Liu et al., 2013). The abundance of penile anaerobes is associated with inflammation and risk of STIs in uncircumcised heterosexual cM (Liu et al., 2013; Prodger et al., 2021); it remains to be investigated if the same is true in the neovagina. It is interesting to note that, based on one study of the TDV (Winston McPherson et al., 2019) and one small study of the neovagina, that the penile skin lined neovagina appears more similar in microbiota composition to the TDV and the uncircumcised penis than to the EDV or the post-menopausal vagina.

**Table 3:** Summary of the characteristics of the estrogen dominated vagina (EDV), post-menopause vagina, testosterone dominated vagina (TDV), sub-preputial penile skin, penile skin-lined neovagina, and sigmoid-lined neovagina.

	Epithelium	Dominant Hormone	Dominant Taxa in Microbiota
**EDV**	Stratified, squamous Thick (~300µm) Glycogen-rich	Estrogen	Optimal: *L. crispatus, L. gasseri, L. jensenii*; commonly dominated by a single bacterial species
			Molecular BV: *G. vaginalis, Ca.* L. vaginae*, A. vaginae, L. iners, Sneathia, Megasphaera, Prevotella, Anaerococcus*
**Post-menopause vagina**	Stratified, squamous Thinner (~150μm) Reduced glycogen	Low estrogen	*L. iners, Streptococcus, G. vaginalis, L. gasseri, Bifidobacterium, Anaerococcus, Corynebacterium, Atopobium, Enterococcus*
**TDV**	Stratified, squamous Thinner (~180μm) Reduced glycogen	Testosterone	*Anaerococcus, Corynebacterium, Finegoldia, Peptoniphilus, Streptococcus, Prevotella, Dialister, Gardnerella, Campylobacter, Fusobacterium, Parvimonas, Porphyromonas* Based on n=28
**Penile skin**	Stratified, squamous Thinner (70-100µm) No glycogen Dense keratin bundles and extracellular lipid deposition	Testosterone	*Prevotella, Peptoniphilus, Porphyromonas, Finegoldia, Anaerococcus, Dialister, Corynebacterium, Murdochiella, Ezakiella, Campylobacter, Negativococcus, Peptostreptococcus, Mobiluncus*
**Penile skin-lined neovagina**	Reduced cornified layer No glycogen	Estrogen	*Prevotella, Peptostreptococcus, Peptoniphilus, Corynebacterium, Porphyromonas*, *Campylobacter* Based on n=4
**Sigmoid colon-lined neovagina**	Unknown *Sigmoid colon is a simple, columnar epithelium with local mucus production*	Estrogen	*Bacteroidaceae, Enterobacteriaceae, Escherichia, Fusobacteriaceae, Actinomycetaceae* Based on n=1

In contrast, the neovaginal microbiome of the only participant who had sigmoid vaginoplasty was clearly distinct, completely lacking *Prevotella* and instead defined by taxa common in the gut microbiota, *Bacteroidaceae* and *Enterobacteriaceae* (Birse et al., 2020). These interesting data suggest that, even many years post-vaginoplasty, the origin of the tissue used defines the colonizing microbiota. It is therefore critically important that all future research of the neovaginal microenvironment consider tissue source, and ideally be powered to afford stratification by tissue source. As a field, researchers and clinicians should be aware that different standards of care and clinical recommendations may be required depending on the tissue used for neovaginal construction.

### Knowledge Gaps

There are several important knowledge gaps in our understanding of the neovaginal microbiome that urgently need to be filled to improve neovaginal healthcare. Preliminary data from Trans PULSE Canada (n=2,873), a national survey of the TGD population in Canada, show nearly half of participants with vaginoplasty experienced gynecological symptoms in the past year, including malodor, abnormal or disturbing discharge, and itching (personal communication, Trans PULSE Canada). Such symptoms are frequently associated with BV in the EDV; however, the underlying cause of these symptoms in the neovagina remains uncharacterized. Neovaginal swabs sent for clinical diagnostics frequently return the results “altered vaginal flora inconsistent with bacterial vaginosis” and treatments established for the EDV (metronidazole) are frequently ineffective (Jain and Bradbeer, 2007; van der Sluis et al., 2020). Candidiasis is another common cause of vaginal itching in the EDV; there has been one case series published reporting neovaginal candidiasis in five individuals (de Haseth et al., 2018), warranting further characterization of neovaginal candida species and the development of clinical guidelines for prevention and treatment. The cause of neovaginal malodour also warrants further investigation. Malodour in the EDV has been associated with the production of biogenic amines by BV-associated bacteria (McMillan et al., 2015; Puebla-Barragan et al., 2021). Larger studies employing omics approaches, including metagenomics, metatranscriptomics and metabolomics to characterize the neovaginal microenvironment in symptomatic individuals are urgently needed to inform treatment options.

Equally important is defining what constitutes an optimal neovaginal microbiota post penile inversion or sigmoid vaginoplasty. This information is essential to guiding treatment options, as what the treatment leaves untouched may be just as important as what it removes. A portion of metronidazole’s efficacy in treating BV of the EDV is that it selectively spares *Lactobacillus*, and thus helps to promote an optimal microbiome that is resistant to re-colonization with inflammatory/pathogenic anaerobes (Petrina et al., 2017). While little is known of the microstructure of the penile-skin lined neovaginal epithelium, if it indeed lacks glycogen and retains cornification, it would be unlikely to promote *Lactobacillus* dominance. A minority of uncircumcised cM [~12% in Uganda (Prodger et al., 2021)] sustain a microbiota under the foreskin fold of the penis that is dominated by *Corynebacterium* with low abundance of Gram-negative anaerobes. This microbiota is associated with low inflammation and reduced risk of STI acquisition (Prodger et al., 2021); future larger studies will reveal if a similar microbiota is optimal in the penile skin-lined neovagina. Additional information on the microstructure of the neovaginal epithelium post penile inversion or sigmoid vaginoplasty would help to inform what type of bacterial commensals might promote an optimal, low-inflammation, protective neovaginal microenvironment.

There is paucity of data on the kind of practices that promote an optimal neovaginal microenvironment, both in the immediate post-operative period and for long-term hygiene and care. Due to a lack of evidence-based guidelines, neovaginal care recommendations vary substantially between centers (Grimstad et al., 2021). Frequent dilation is necessary post-operatively to prevent stenosis of the neovaginal canal (Goddard et al., 2007; Horbach et al., 2015; Buncamper et al., 2016; Loree et al., 2020); most centers recommend at least two dilations a day for the first six months decreasing to once weekly after a year (Buncamper et al., 2016). Ample water-based lubrication is recommended to increase the ease of the dilations and to protect the integrity of the surgical dilators. Frequently the use of a vaginal douche after dilation is recommended with varied solutions including water, soap, vinegar, or povidone iodine solutions (Goddard et al., 2007; Deutsch, 2016; Pan et al., 2019). Douching and the use of soaps or lubricants can promote molecular BV in the EDV of cF, but their effects on the neovaginal microbiota are unknown. Regular use of hygienic products and even boric acid [to lower the pH, promote colonization with *Lactobacillus*, and treat vaginal yeast infection (Donders et al., 2010; Iavazzo et al., 2011)] are also frequently reported, however, such efforts would be in vain, and potentially disruptive, if the optimal neovaginal microbiome is found to be dominated by *Corynebacterium* (penile skin-lined) or *Bacteroidaceae* (sigmoid-lined).

## Conclusions

As access to gender-affirming hormone therapy and surgery increases, a growing number of TGD persons will need access to effective and evidence-informed gynecological care. There is an urgent and growing need to identify the causative agents of the unique gynecological concerns of TGD populations and to define clinical guidelines to promote gynecological health. In tM individuals on testosterone therapy, vaginal pain, bleeding, atrophy, and non-*Lactobacillus*-dominated vaginal microbiota are common. Further research is warranted to establish the role of testosterone augmentation beyond that of estrogen deprivation. Anecdotal evidence suggests topical estrogen therapy may promote a *Lactobacillus-*dominated microbiome, justifying further studies to investigate if this approach can alleviate symptoms. In tF individuals with a neovagina, gynecological symptoms such as abnormal discharge, itching and malodor are common, but the etiology of these symptoms remains unknown, and treatments designed for cF may be ineffective. The limited data we have of the neovaginal microbiome (n=5) suggests that it is very unlike that of reproductive-aged or post-menopausal cF and may have more commonalities with the microbiota of the uncircumcised penis of cM or the vagina of tM on testosterone therapy. Importantly, the limited available data suggests the tissue used to create the vaginal canal may have a substantial impact on the subsequent microbiota and should be considered and reported in future research. What defines optimal vs. non-optimal microbiota in different types of neovaginas, and what bacteria are pathogenic, is yet to be defined and this information is critically needed to improve clinical management and treatment options.

## Author Contributions

JP, JR, GB, YK, EP, HW, JH, and BM contributed to literature review, manuscript writing, and editing. All authors contributed to the article and approved the submitted version.

## Funding

JP was supported by a Canada Research Chair (Canadian Institutes of Health Research: 950-233211); funds from the Schulich School of Medicine and Dentistry, Western University; and the National Institute for Allergy and Infectious Diseases of the National Institutes of Health (R01AI123002). GB is supported by a Canadian Institutes of Health Research Sex and Gender Science Chair (GSB-171372). JR and BM were supported by the National Institute for Allergy and Infectious Diseases of the National Institutes of Health (R01NR015495)

## Conflict of Interest

The authors declare that the research was conducted in the absence of any commercial or financial relationships that could be construed as a potential conflict of interest.

## Publisher’s Note

All claims expressed in this article are solely those of the authors and do not necessarily represent those of their affiliated organizations, or those of the publisher, the editors and the reviewers. Any product that may be evaluated in this article, or claim that may be made by its manufacturer, is not guaranteed or endorsed by the publisher.
